# Climatic Niche Shift during *Azolla filiculoides* Invasion and Its Potential Distribution under Future Scenarios

**DOI:** 10.3390/plants8100424

**Published:** 2019-10-18

**Authors:** Argantonio Rodríguez-Merino, Rocío Fernández-Zamudio, Pablo García-Murillo, Jesús Muñoz

**Affiliations:** 1 Department of Plant Biology and Ecology, Faculty of Pharmacy, University of Seville, Profesor García González 2, 41012 Seville, Spain; pgarcia@us.es; 2 Doñana Biological Station (EBD-CSIC), Avda. Américo Vespucio s/n, 41092 Seville, Spain; rzamudio@ebd.csic.es; 3 Real Jardín Botánico (RJB-CSIC), Plaza de Murillo 2, 28014 Madrid, Spain; jmunoz@rjb.csic.es

**Keywords:** aquatic plants, climate change, invasive species, MaxEnt, niche overlap, species distribution models

## Abstract

In order to prevent future biological invasions, it is crucial to know non-native species distributions. We evaluated the potential global distribution of *Azolla filiculoides*, a free-floating macrophyte native to the Americas by using species distribution models and niche equivalency tests to analyze the degree of niche overlap between the native and invaded ranges of the species. The models were projected under two future emission scenarios, three global circulation models and two time periods. Our results indicate a possible niche shift between the distribution ranges of the species, indicating that *A. filiculoides* can adapt to novel environmental conditions derived from climatic differences during the invasion process. Our models also show that the future potential distribution of *A. filiculoides* will decrease globally, although the species could colonize new vulnerable regions where it is currently absent. We highlight that species occurrence records in the invaded area are necessary to generate accurate models, which will, in turn, improve our ability to predict potential invasion risk areas.

## 1. Introduction

Biological invasions and climate change are among the top five major drivers of biodiversity loss in freshwater ecosystems, along with land-use change, nitrogen deposition, and elevated carbon dioxide concentrations [1,2]. The effects of climate change can dramatically influence the distribution of aquatic species, including non-native ones [3,4]. Knowing the potential distribution of invasive species would be decisive not only for minimizing damage associated with accidental introductions, but also for the development of early detection programs and strategies in susceptible areas [5,6,7]. In addition, in order to understand the future potential distribution of these species under the effects of projected climate scenarios, it is crucial to identify current species dynamics, which can be the basis for implementing appropriate management actions to reduce negative consequences on natural habitats and biodiversity [3].

The invasion process is more successful when species colonize climatic environments similar to those of its native range [8]. The niche conservatism principle (niches are conserved through space and time) is, therefore, often considered in analyses of non-native species potential distribution. Under this principle, niche models are calibrated on the native distribution area and projected onto the invaded one to assess invasion risk [8,9]. However, there are many factors that influence the invasion process, such as propagule pressure, geographic and/or climatic barriers, human activities, competition between organisms, as well as many other factors that can result in species niche divergence [10,11]. Analyses of the potential distribution of species that ignore non-native species occurrences in non-native regions could produce uncertainties, as all of the environmental variability acquired by the studied species in novel colonized habitats would not be taken into account [11,12].

In recent years, species distribution models (SDMs) have become more frequently used in the management and control of non-native species [13,14,15]. SDM techniques allow correlating species occurrence records with environmental variables to uncover the potential distribution of a species and, thus, to find the most suitable areas for the species survival [14,16]. For this reason, SDMs have been used in some studies to analyze whether there is niche conservatism or niche shift between native and invaded distribution ranges, the results of which were used to design appropriate management strategies [11,12].

Aquatic invasive plants are considered among the most damaging invaders in freshwater ecosystems as they can alter the structure and composition of habitats, and the biogeochemistry and water quality of the systems they invade [17]. Principal characteristics that allow aquatic plants to be potential invaders include phenotypic plasticity and their ability to tolerate a wide range of environmental conditions and to adapt to disturbed systems [15,18]. Changes in species physiology due to phenotypic plasticity together with propagule pressure, favored mainly by human activities, can lead to the use of different niches by non-native aquatic plants [9,19]. These characteristics make aquatic plants excellent model organisms to study environmental niche changes.

**Figure 1:**
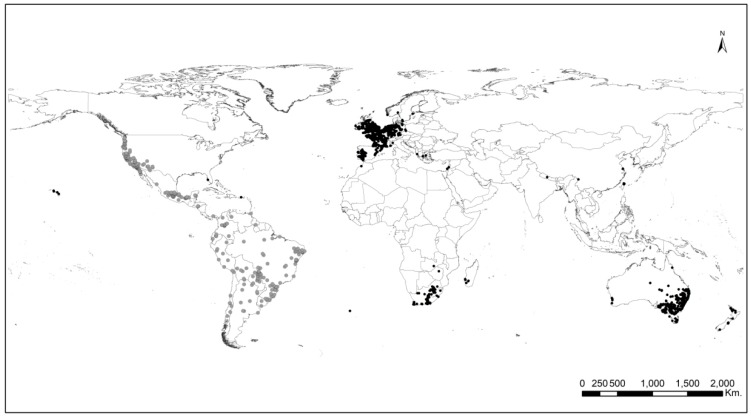
Global geographic distribution of *Azolla filiculoides*. Grey dots represent native occurrence localities and black dots represent invaded occurrence localities.

Our study focuses on *Azolla filiculoides* Lamarck 1783 (Salviniaceae), a small heterosporous free-floating fern native to the American continent [20] that is now distributed worldwide (Figure 1) and is considered an aquatic invasive species (AIS) in some geographic areas [21]. *Azolla filiculoides* has a very high growth rate, even under suboptimal conditions, and it can form dense mats that block light, leading to the impoverishment or even disappearance of submerged plants and associated communities [22,23]. In addition, this species forms a symbiosis with a nitrogen-fixing cyanobacteria to obtain atmospheric nitrogen [24,25], and its potential for vegetative reproduction in the absence of adequate conditions is very high [26]. Given these features, *A. filiculoides* is one of the most dangerous invaders affecting freshwater habitats and their biodiversity [27,28,29,30].

Here, we used SDMs to investigate a possible niche shift during the *A. filiculoides* invasion process. We also used two metrics of niche overlap and multivariate techniques to further support the SDM results [12,31,32]. Our main aims were to (1) examine whether the climatic niche of *A. filiculoides* is uniform between native and invaded ranges, (2) characterize the climatic tolerances of *A. filiculoides* in both native and invaded ranges and (3) describe the future potential distribution of *A. filiculoides* at the global scale.

## 2. Results

### 2.1. Niche Overlap in Environmental Space

**Figure 2:**
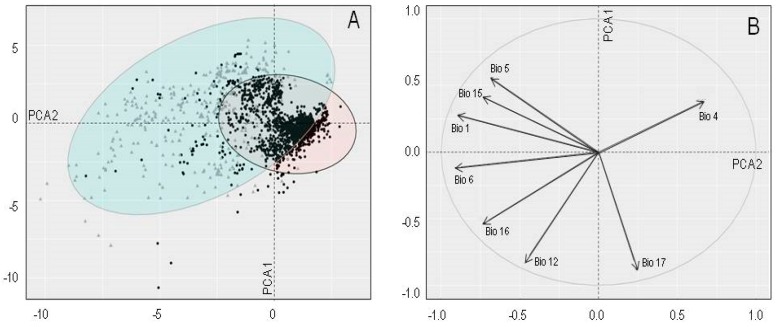
(**A**) The principal component analysis (PCA) for two *Azolla filiculoides* ranges. Grey triangles represent native localities and black dots represent invasive localities in the environmental space. Ellipse level 0.95. (**B**) Bioclimatic variables in the environmental space defined by the first two principal component axes. PCA1 and PCA2 explained 48.8% and 31% of the variation in the data, respectively. Annual mean temperature (Bio 1), temperature seasonality (Bio 4), maximum temperature of the warmest month (Bio 5), minimum temperature of the coldest month (Bio 6), annual precipitation (Bio 12), precipitation seasonality (Bio 15), precipitation of the wettest quarter (Bio 16), and precipitation of the driest quarter (Bio 17).

The native and invasive distributions of *A. filiculoides* are clearly separated in multidimensional environmental space (Figure 2A). The first two Principal Component Analysis (PCA) axes explained 79.8% of the variation in the data (PCA1: 48.8% and PCA2: 31%). The first PCA axis was positively related to temperature seasonality (Bio 4) and precipitation of the driest quarter (Bio 17), whereas the second PCA axis was negatively related to minimum temperature of the coldest month (Bio 6), annual precipitation (Bio 12), precipitation of the wettest quarter (Bio 16) and Bio 17 (Figure 2B). The non-native occurrence records occupied drier areas with higher pronounced seasonality. There are some climatic coincidences in the distribution ranges, although the invaded range shows new climatic areas unknown in the species’ native distribution range (Figure 2A).

### 2.2. Species Distribution Modeling

**Figure 3:**
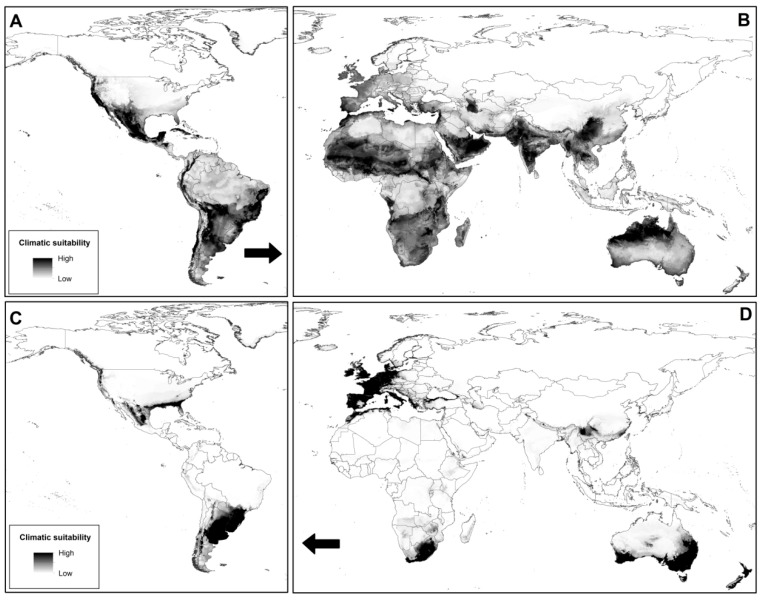
Current potential distribution of *Azolla filiculoides* based on reciprocal models. (**A**) Model calibrated with native occurrences and (**B**) model projected onto the invasive range. (**C**) Model projected onto the native range and (**D**) model calibrated with invasive occurrences. Darker shades indicate higher environmental suitability. Arrows indicate the direction of model projections.

**Figure 4:**
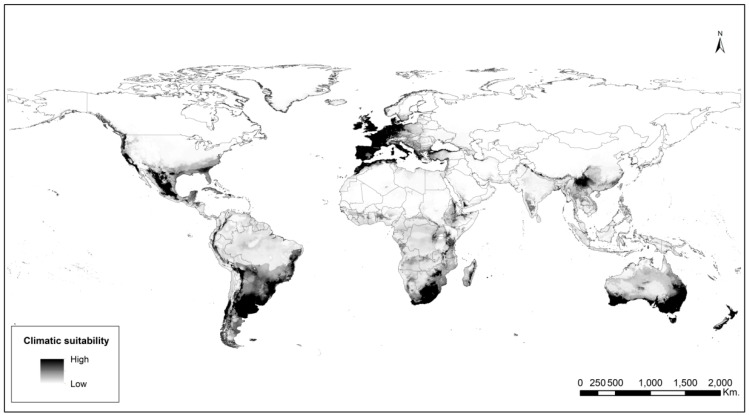
Current distribution and climate suitability model of *Azolla filiculoides* based on presence occurrences from both invaded and native ranges. Darker shades indicate higher environmental suitability.

The high area under the receiver operating characteristic (ROC) curve AUC values indicated highly accurate performance for all models (Table 1). The native model (trained on the native area) reflected the current distribution of *A. filiculoides* in the Americas (Figure 3A). However, when the native model was projected onto the invaded area, the predicted potential distribution was broader than the known distribution of the species (Figure 3B). In contrast, the invasive model (trained on the invaded area) underestimated the suitable area of *A. filiculoides* on the native region (Figure 3C), though it correctly showed the distribution of the species on the invaded range (Figure 3D). Figure 4 shows a suitability map for *A. filiculoides* under current climatic conditions using native and invaded range occurrences.

### 2.3. Niche Overlap Analyses

The results of the identity test allowed us to reject the null hypothesis of niche identity, since Schoener’s *D* (0.401) and Hellinger’s *I* statistics (0.703) were lower than expected by chance (Figure S1, Supplementary Materials). The background similarity test results proved that niche similarity was greater than expected by chance in both directions, i.e., from native to invaded range and from invaded to native range (*D*: 0.405 and *I*: 0.705; Figure S2).

### 2.4. Predicted Niche Occupancy

**Figure 5:**
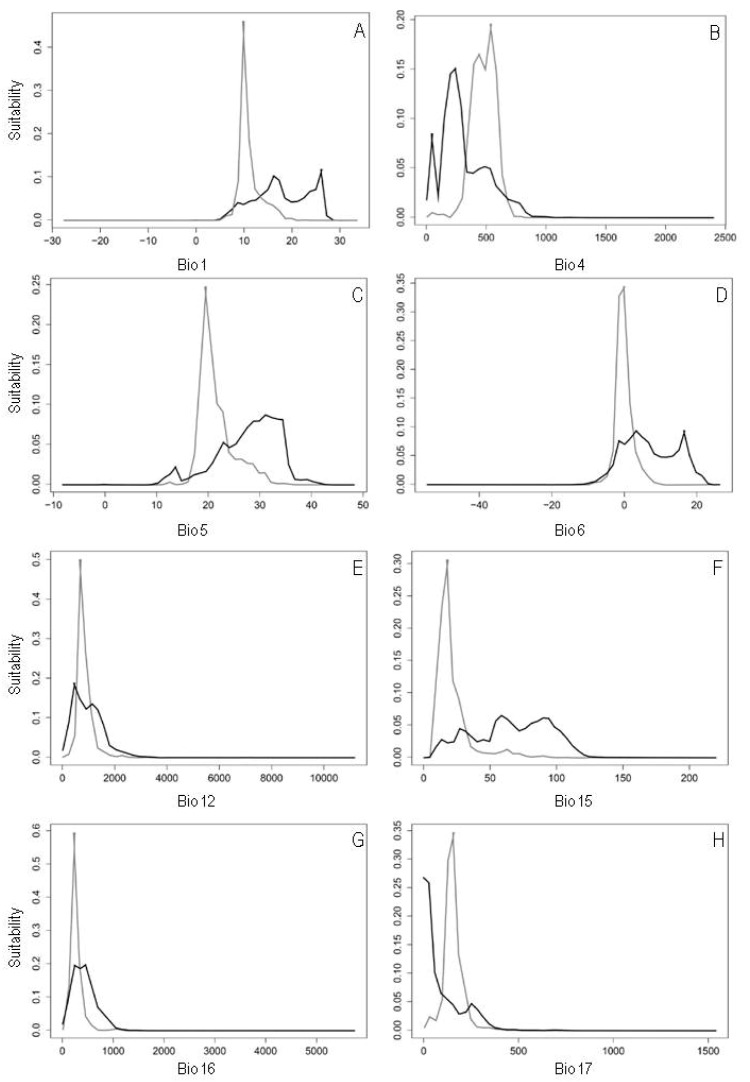
Predicted niche occupancy (PNO) profiles. The vertical axes show the total suitability of the bioclimatic variable and the horizontal axes show the bioclimatic variable for each range over its geographic distribution. The overlapping zones indicate similar climatic tolerances, and the amplitude of the profile indicates the specificity of climatic tolerance. Black lines represent the native range of *Azolla filiculoides*, and grey lines the invasive range of *Azolla filiculoides*. (**A**) Annual mean temperature (Bio 1), (**B**) temperature seasonality (Bio 4), (**C**) maximum temperature of the warmest month (Bio 5), (**D**) minimum temperature of the coldest month (Bio 6), (**E**) annual precipitation (Bio 12), (**F**) precipitation seasonality (Bio 15), (**G**) precipitation of the wettest quarter (Bio 16), and (**H**) precipitation of the driest quarter (Bio 17).

Predicted niche occupancy (PNO) profiles indicated differences in the climatic requirements of *A. filiculoides* in native versus invaded ranges (Figure 5). The potential distribution of *A. filiculoides* in the invaded range showed greater suitability in colder areas (Figure 5A,C,D), with more pronounced temperature seasonality (Figure 5B), lower precipitation seasonality (Figure 5F) and drier summers (Figure 5H). On the contrary, the potential distribution in the native range noted greater suitability in warmer environments (Figure 5A,C,D) that have lower temperature seasonality (Figure 5B), and higher and more pronounced seasonal precipitation (Figure 5E–G).

### 2.5. Potential Distribution Comparisons Under Future Scenarios

**Figure 6:**
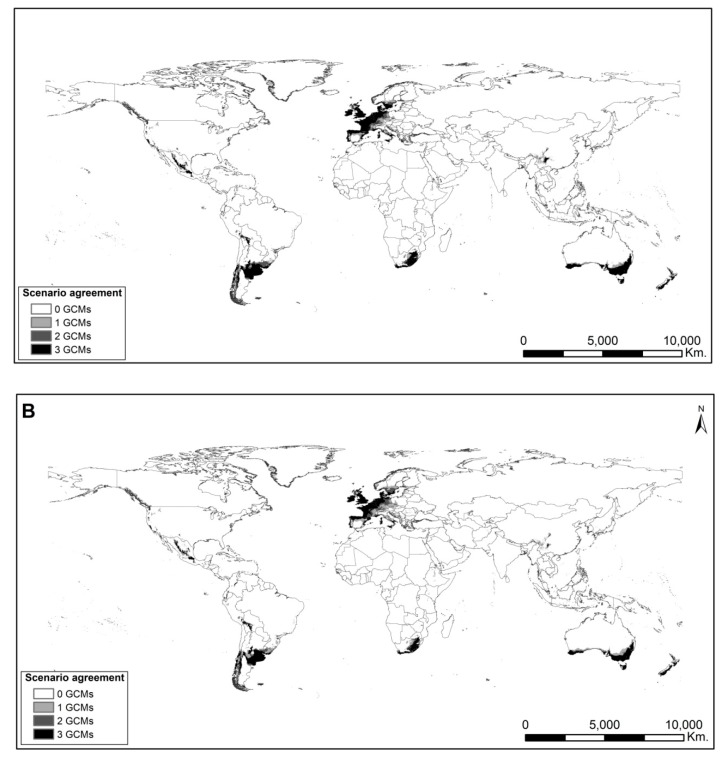
Projected potential distribution of *Azolla filiculoides* under the optimistic emission scenario (RCP 4.5) and three different GCMs. (**A**) 2050. (**B**) 2080. Darker shades indicate greater overlapping of GCMs.

When the models were considered under future emission scenarios, a dramatic reduction in the potential distribution of *A. filiculoides*, especially in the native area, was observed (Figure 6 and Figure S3). This reduction was more pronounced under the pessimistic emission scenario (RCP 8.5; Figure S3). The overlap of global circulation models (GCMs) was high, especially under the optimistic emission scenario (RCP 4.5; Figure S4). In contrast, under the pessimistic emission scenario (RCP 8.5), and mainly for the later temporal period (2080), the models showed greater variability in terms of predicted projections (Figures S3 and S4). Additionally, according to the models, *A. filiculoides* becomes displaced towards the poles in the invaded areas (Figure 6 and Figure S3). Of particular importance is the increasing suitability towards eastern and northern Europe, where the species is currently unknown or exceedingly rare but could colonize and spread to further climatically suitable areas (Figure 6 and Figure S3).

## 3. Discussion

Our findings demonstrate that the climatic niche of *A. filiculoides* in both ranges, native and invaded, is similar but not identical. According to the identity test results, niche overlap was less than expected by the null model. Therefore, the identity null hypothesis was rejected, suggesting that native and invaded niches are environmentally different [31]. On the other hand, the background similarity test results indicated that the niches are more similar than expected by chance and, thus, the null hypothesis of background similarity could not be rejected [31]. Taken together, our results indicate that *A. filiculoides* shares environmental niche spaces in both distribution ranges, presenting similar climatic requirements. However, different environmental variables restrict its potential distribution in the two areas, and new suitable environments appear to be available for *A. filiculoides* in the invaded range, suggesting an environmental niche shift between native and invaded areas.

Several factors can induce a niche shift during the invasion process, which, in turn, can facilitate colonization of an area with novel combinations of environmental conditions, leading a species to exceed the limits of its native niche and to occupy different non-native niches [33]. These factors include abiotic differences, biotic interactions, dispersive barriers or human activities [11]. Climatic differences can play an important role in ecological divergence processes [34,35] by, promoting the adaptation of species to new climatic environments [36]. Our models support the cosmopolitan distribution of *A. filiculoides* and, as demonstrated by the PNO profiles, indicate that the species is able to survive in a wide spectrum of environmental conditions. In addition, the low niche conservatism of *A. filiculoides* makes it a very dangerous invaders of freshwater ecosystems as, it is able to survive in a wide variety of habitats, including extreme climatic environments, such as in Great Britain, where the species survives in very low temperatures compared to those of its native range [22].

The effect of human activity also significantly influences the invasion processes of non-native species [37]. Indeed, Rodríguez-Merino et al. [38,39] previously described how the human footprint (a variable that represents the impact of humans on a territory) is a key variable in the potential distribution of *A. filiculoides*. In this context, changes in watersheds by human activities (e.g.,: agricultural, industrial) can affect aquatic species distribution [40], causing habitat loss for native species and, consequently, the occupation of empty habitats by non-native species (empty niche hypothesis [41]). Additionally, the access to available resources is one of the major drivers controlling the invasion process (fluctuating resources theory [42]). Another important aspect to consider is role of potential competitors that can regulate non-native species populations by, limiting their dispersion in the invaded range (enemy release hypothesis [41]). The absence/presence of competitors and predators changes the environmental conditions of an invaded area [11]. This has been demonstrated for *A. filiculoides* in the invaded range: the species is in decline or has disappeared at sites in which its natural enemy, the weevil *Stenopelmus rufinasus*, is found [43]. For example, in Doñana, *A. filiculoides* has disappeared from ponds with *S. rufinasus* but thrives in nearby water bodies uninhabited by the weevil. The influence of these factors together with highly successful clonal growth [26] and the symbiosis with its cyanobiont [25], could imply changes in the *A. filiculoides* invasive niche during the invasion process.

The models under future projections suggest that *A. filiculoides* could colonize new areas outside its native range where it is still absent. In this context, there is a large proportion of aquatic environments susceptible to being colonized by the species. Changes in the future potential distribution of *A. filiculoides* were more pronounced under higher emission concentration (pessimistic) scenario (RCP 8.5), particularly in Europe where the species distribution is projected to have a northward latitudinal trend. However, the effects of climate change will not only influence the species’ geographic trends (latitudinal and/or altitudinal) but also its potential distribution area, which will be reduced. These results coincide with those proposed for other AIS in Europe [4]. From the perspective of *A. filiculoides* invasion management and control, the reduction of its non-native potential distribution area is good news. However, the effects of climate change will also transform aquatic ecosystem conditions, which will, in turn, influence the distribution of native aquatic vegetation through the potential loss of available habitats and consequent loss of populations [44]. In addition, *A. filiculoides* shows an alarming reduction of potential distribution in its native area, caused by the loss of suitable habitats in some regions, a trend that is observed for other AIS [4].

Increasing water temperatures and water regime alterations caused by changes in precipitation could cause displacements in the species distribution areas, including the non-native area [3]. In the case of *A. filiculoides*, increasing temperatures and, consequently, environmental aridification could lead to the loss of available habitats. However, new area gains towards high latitudes, particularly in northern Europe, have been detected, as our analyses suggest. This demonstrates that new habitats are available for *A. filiculoides*. On the other hand, the decrease and change in rainfall frequency could influence aspects related to connectivity and the dispersion of the species towards new environments [4].

Our results indicate that niche equivalence analyses provide crucial information for SDM projections, allowing the potential distribution of non-native species to be more accurately assessed. Similar results have been proposed for other AIS [19]. The high AUC values showed that the reciprocal models performed very well, although they are not adequate to determine the species potential distribution onto the projected range, especially when the transfer is from the invaded onto the native area. In this context, the combination of species occurrence data (native and invaded localities) can substantially improve the accuracy of the potential distribution prediction [11]. However, the use of these methods is not sufficient to detect whether the change produced in the species environmental niche between native and invaded ranges is caused by a truly adaptive process, by a change in propagule pressure success or even by historical contingency process [9]. Regarding this, it would be interesting to perform future studies related to the physiological aspects of *A. filiculoides*, to assess its adaptive capacity in the face of different stress factors in order to determine which are the main drivers that explain the niche shift during the invasion process.

## 4. Materials and Methods

### 4.1. Occurrence Records

An *A. filiculoides* occurrence records database was compiled from three sources: Global Biodiversity Information Facility (GBIF, http://www.gbif.org/; https://doi.org/10.15468/dl.wph41n), Species Link (http://splink.cria.org.br/) and TROPICOS (http://www.tropicos.org/). Occurrence records from native area were checked using standard floras (Supplementary Materials, Annex S1). To minimize overfitting due to the potential negative effect of spatial autocorrelation [45], the occurrence records were limited to one per pixel of environmental layers.

### 4.2. Bioclimatic Variables

Bioclimatic variables used for building the models were previously described by Fick and Hijmans [46] and were obtained from the WorldClim database (Table S1, http://www.worldclim.org/version2), at 5-arc minute spatial resolution (~10 km at the Equator). The bioclimatic variables describe the average, the variation (seasonality) and the extreme values of temperature and precipitation. These variables induce physiological stress in plants and are useful to limit the distribution of aquatic plants [47]. The number of environmental variables used to fit models was reduced to minimize model overfitting [48]. Eight variables were selected: annual mean temperature (Bio 1), temperature seasonality (Bio 4), maximum temperature of the warmest month (Bio 5), minimum temperature of the coldest month (Bio 6), annual precipitation (Bio 12), precipitation seasonality (Bio 15), precipitation of the wettest quarter (Bio 16) and precipitation of the driest quarter (Bio 17). These bioclimatic variables have been shown to be useful for studying the potential distribution of aquatic plants at large scales and for establishing the potential geographic limits of species and the climatic variability to which aquatic plants are exposed [4,47,49]. To obtain the best possible models, we did not eliminate correlated variables as the maximum entropy algorithm (see species distribution modeling section) makes robust predictions even when variables are correlated [50,51]. This capacity allows the algorithm to choose the most explanatory variables among all variables included in the species distribution modeling [50,52].

The future potential distribution of *A. filiculoides* was projected for two future emission scenarios, one optimistic and the other pessimistic, using the representative concentration pathways (RCPs: 4.5 and 8.5, respectively) [53], and for two time periods (2050 and 2080) using three global circulation models (GCMs): CSIRO MK 3_6_0, MIROC MIROC 5 and MOHC HADGEM 2 ES. The use of three GCMs accounts for the uncertainty associated with such models [54]. These data were obtained from the CGIAR Research Program on Climate Change, Agriculture and Food Security (http://www.ccafs-climate.org).

### 4.3. Niche Overlap in Environmental Space

Principal Component Analysis (PCA) was used to quantify differences between native and invaded niches in multidimensional environmental space. The *A. filiculoides* occurrence records were plotted against eight selected bioclimatic variables to obtain a two-dimensional climatic range summarized by the first two axes of the PCA [12]. PCA analyses were run using the ‘ggfortify’ package [55] and the data were visualized using the ‘ggplot2’ package [56] implemented in R software v3.1.2 [57].

### 4.4. Species Distribution Modeling

Species distribution models (SDMs) were used to establish the geographic pattern of *A. filiculoides* and to compare environmental niche differences between native and invaded areas. SDMs were run under the maximum entropy model implemented in the MaxEnt software v.3.3.3k [58]. The MaxEnt model has proven to perform better than other modeling methods and is one of the most effective presence-only data algorithms currently available [59,60,61,62]. MaxEnt models were generated by default parameters (‘Auto features’, convergence = 10^−5^, maximum number of iterations = 500, prevalence = 0.5, regularization value β = 1) [50,59,63]. The MaxEnt algorithm requires the use of background points: these are random points that represent the calibration area, which typify the environmental conditions available for the species [50]. Three background datasets of 10,000 points each were generated: (a) in the global model, the points were selected from all suitable lands except Antarctica, the only area where *A. filiculoides* does not grow; (b) in the native model (see reciprocal niche models section), the points were selected from throughout the Americas; and (c) in the invasive model, they were selected from all areas where *A. filiculoides* grow, excluding the Americas and Antarctica. Ten replicates were run for each model to estimate the associated error with the fitted function using 70% of the presence to train the model, and the remaining 30% to test the model [50]. The area under the receiver operating characteristic (ROC) curve, or AUC value, was used to evaluate model accuracy. The AUC is the most popular metric to evaluate MaxEnt models [51]. Continuous suitability maps were transformed into binary maps (presence/absence) using the 10th percentile training presence threshold [39,64].

### 4.5. Reciprocal Niche Models

Geographical niche shifts during the invasion process were studied by reciprocal models. A model with only native occurrences was first run and then projected onto the invasive area (native model). A second model with only invaded occurrences was then run and projected onto the native area (invasive model). A final model run was with both native and invasive occurrences (global model) [12,65].

### 4.6. Niche Overlap Analyses

To estimate niche overlap, Schoener’s *D* and a modified Hellinger distance’s *I* statistics were calculated using the ‘phyloclim’ package implemented in R [66]. The values of the two metrics range from 0 (no niche overlap) to 1 (total niche overlap). The classification proposed by Rödder and Engler [67] was followed to facilitate the interpretation of the degree niche overlap. These measures were used to test niche identity (or niche equivalence) and background similarity between native and invaded ranges in the geographical space [31]. The niche identity test determines whether the niches observed in both geographic ranges (native and invaded) are more equivalent than expected by chance when the occurrences of both distributions (native and invasive) are reassigned between the two analyzed ranges. The background similarity test determines whether the overlap between occupied niches can be attributed to the available environmental space [68]. These metrics are commonly used to compare geographical models [69]. For both tests, 100 randomizations were performed to produce the null distributions [31].

### 4.7. Predicted Niche Occupancy

Predicted niche occupancy (PNO) profiles quantify the multimodal variation in bioclimatic parameters through species probability distribution obtained by the SDMs [70]. The PNO profiles were used to estimate the climatic tolerances of *A. filiculoides*, in its native and invasive ranges [36]. The PNO analyses were built in the ‘phyloclim’ package implemented in R [66], using MaxEnt raw outputs and 50 equally spaced bins spanning the parameter range of each bioclimatic variable [71].

### 4.8. Potential Distribution Comparisons Under Future Scenarios

To address the effects of climate change on the future potential distribution of *A. filiculoides*, global binary models were used to quantify the predicted suitable area. Three binary maps corresponding to three GCMs were stacked for each climatic emission scenario (RCPs: 4.5 and 8.5) and for each studied time period (2050 and 2080) in order to quantify potential distribution coincidences between GCMs.

## 5. Conclusions

The prevention and the control of biological invasions are crucial aspects to consider for the maintenance of ecosystemic stability, particularly given that invasive species can negatively impact ecosystems [33]. Our results provide useful insights to anticipate the presence of *A. filiculoides* in areas of high invasion risk, on the basis of its bioclimatic requirements, as well as to establish conservation strategies for the species in its native area. We consider our approach for determining possible divergences in both distribution ranges very useful for non-native aquatic plants, since they can alter their physiology in response to novel conditions in the invaded range due to their high plasticity, which could allow future species niche evolution [9]. Considering this, the use of species occurrence records in the native and invaded ranges allows us to develop more precise geographic models that include the environmental variability acquired by the species in newly colonized ranges. Taken together, this information can serve as a powerful resource to help decision makers establish early warning systems to mitigate the effects of future invasions of *A. filiculoides* under climate change pressures. In addition, this protocol can be applied to other invasive species in different ecosystems.

## Figures and Tables

**Table 1:** The table shows the number of occurrence records (N) was used for building the different models and the AUC values and standard deviation for *Azolla filiculoides* models performance.

	Native Model	Invasive Model	Global Model
N (occurrence records)	331	1882	2213
AUC ± SD	0.907 ± 0.023	0.916 ± 0.004	0.895 ± 0.006

## Supplementary Materials

The following are available online at https://www.mdpi.com/2223-7747/8/10/424/s1, Annex S1: References list of traditional floras to establish the native area of *Azolla filiculoides*, Table S1: Description of bioclimatic variables according to WorldClim available at http://www.worldclim.org/version2, Figure S1: The graphs show the niche identity test, showing Schoener’s *D* and modified Hellinger distance’s *I*. The observed similarity between niches is indicated with the line, while bars indicate the null distribution of ecological niche distances generated randomly. Shoener’s *D* and Hellinger distance’s *I* range from 0 (completely different), to 1 (identical), Figure S2: The graphs show the background similarity test, showing Schoener’s *D* and modified Hellinger distance’s *I*. The observed similarity between niches is indicated with the line, while bars indicate the null distribution of ecological niche distances generated randomly. Shoener’s *D* and Hellinger distance’s *I* range from 0 (completely different), to 1 (identical), Figure S3: Projected potential distribution of *Azolla filiculoides* under the pessimistic emission scenario (RCP 8.5), corresponding to three different GCMs. (A) 2050. (B) 2080. Darker shades indicate greater overlapping of GCMs, Figure S4: Number of total pixels (1 pixel ~ 10km of resolution) predicted as suitable under each GCM projection. The data corresponds to the sum of the binary models. Overlap values: 1 GCM (no model overlap), 2 GCMs (two models overlap) and 3 GCMs (three models overlap).
