# Supplementary material for: Climatic Niche Shift during Azolla filiculoides Invasion and Its Potential Distribution under Future Scenarios
Source: Plants (Basel). 2019 Oct 18;8(10):424. doi: 10.3390/plants8100424 (PMC6843849; doi:10.3390/plants8100424)
Supplement: Supplementary file 1 [file plants-08-00424-s001.zip › Supplementary_Materials_Plants.docx]

**Supplementary Materials**

Article

Climatic niche shift during *Azolla filiculoides* invasion and future potential distribution

Argantonio Rodríguez-Merino 1*, Rocío Fernández-Zamudio 2, Pablo García-Murillo 1and Jesús Muñoz 3

^1 Department of Plant Biology and Ecology, Faculty of Pharmacy, University of Seville, Profesor García González, 2, 41012 Seville, Spain.^

^2 Doñana Biological Station (EBD-CSIC), Avda. Américo Vespucio, s/n, 41092 Seville, Spain.^

^3 Real Jardín Botánico (RJB-CSIC), Plaza de Murillo, 2, 28014 Madrid, Spain.^

^* Correspondence: Argantonio Rodríguez-Merino. Department of Plant Biology and Ecology, Faculty of Pharmacy, University of Seville, Profesor García González, 2, 41012 Seville, Spain. E-mail: argantonio.rodriguez@gmail.com^

**Annex S1.** References list of traditional floras to establish the native area of *Azolla filiculoides*.

1. America FON, 1987. Flora of North America Newsletter. St. Louis, Miss. EE.UU.

2. Cavalcanti, T.B., & E.B.A. Dias, 2002. Flora do Distrito Federal, Brasil. Embrapa Recursos Genéticos e Biotecnologia.

3. Correa, M.N., M. Barros, E.G.C. Nicora, & L. Angel, 1969. Flora patagónica. Instituto Nacional de Tecnología Agropecuaria (Argentina).

4. Crow, G.E., & C.B. Hellquist, 2000. Aquatic and wetland plants of northeastern North America: vol. 1. Pteridophytes, gymnosperms and angiosperms, dicotyledons. Madison, Wis.: University of Wisconsin Press 480p.-illus.. ISBN 029916330x En Icones, Keys. Geog 3.

5. Davidse, G., M.S. Sánchez, & S. Knapp, 1995. Flora Mesoamericana: Psilotaceae a Salviniaceae. UNAM.

6. Forzza, R.C., P. M. Leitman, & A. Costa, 2010. Catálogo de plantas e fungos do Brasil-Vol. 1. JBRJ.

7. Gunckel, H., 1984. Helechos de Chile. Ediciones de la Universidad de Chile, Santiago, Chile.

8. Hokche, O., P. Berry, & O. Huber, 2008. Nuevo Catálogo de la Flora Vascular de Venezuela, Fundación Instituto Botánico de Venezuela “Dr. T. Lasser”. Caracas.

9. Jørgensen, P.M., & S. León-Yánez, 1999. Catalogue of the vascular plants of Ecuador. Missouri Botanical Garden Press St. Louis.

10. Jørgensen, P.M., M.H. Nee, S.G. Beck, S. Arrázola, & M. Saldias, 2014. Catálogo de las plantas vasculares de Bolivia. Missouri Botanical Garden Press.

11. Knobloch, I.W., & D.S. Correll, 1962. Ferns and fern allies of Chihuahua, Mexico.

12. Lasser, T., 1964. Flora de Venezuela, vol. 1-15. Instituto Botánico, Caracas.

13. Leiva Sánchez, A., P.P. Álvarez, & D.A. Puentes, 2002. Flora de la República de Cuba. Serie A. Plantas Vasculares. Fascículo 6.

14. Morrone, O., & M.J. Belgrano, 2008. Catálogo de las plantas vasculares del Cono Sur (Argentina, S de Brasil, Chile, Paraguay y Uruguay). Monogr. Syst. Bot. Missouri Bot. Gard 107, 45-48.

15. Murillo-Pulido, M.T., J. Murillo-Aldana, A. León-Parra, & L.A. Triana-Moreno, 2008. Los pteridofitos de Colombia. Universidad nacional de Colombia. Facultad de ciencias. Instituto de ciencias naturales.

16. Reiche, K.F., 1905. Flora de Chile. impr. Cervantes.

17. Reitz, R., 1970. Flora ilustrada catarinense. Herbario Barbosa Rodrigues.

18. Rzedowski, J., G.C. de Rzedowski, C.R. del Bajío, & M. Pátzcuaro, 1968. Flora del Bajío y de regiones adyacentes. America 20, 107-135.

19. Sosa, V., & A. Gómez-Pompa, 1998. Flora de Veracruz. INIREB.

20. Stevens, W.D., C. Ulloa, A. Pool, & O.M. Montiel, 2001. Flora de Nicaragua. Missouri Botanical Garden Press Louis eMissouri Missouri.

21. Stolze, R.G., 1976. Ferns and fern allies of Guatemala: part 1. Ophioglossaceae through Cyatheaceae.: Fieldiana, Bot 39.

22. Thomas, J.H., 1961. Flora of the Santa Cruz Mountains of California: a manual of the vascular plants. Stanford University Press.

23. Wiggins, I.L., 1980. Flora of Baja California. Stanford University Press.

24. Zuloaga, F.O., & O. Morrone, 1996. Pteridophyta, Gymnospermae y Angiospermae (Monocotyledoneae). Catálogo de las Plantas Vasculares de la República Argentina I. (FO Zuloaga & O. Morrone, eds.). Missouri Botanical Garden Press, St. Louis.

**Table S1.** Description of bioclimatic variables according to WorldClim available at http://www.worldclim.org/version2.

| **Variables** | **Description** |
| --- | --- |
| Bio 1 | Annual mean temperature |
| Bio 2 | Mean diurnal range (mean of monthly (maximum temperature – minimum temperature)) |
| Bio 3 | Isothermality (Bio 2/ Bio 7) * 100 |
| Bio 4 | Temperature seasonality (standard deviation * 100) |
| Bio 5 | Maximum temperature of warmest month |
| Bio 6 | Minimum temperature of coldest month |
| Bio 7 | Temperature anual range (Bio 5- Bio 6) |
| Bio 8 | Mean temperature of wettest quarter |
| Bio 9 | Mean temperature of driest quarter |
| Bio 10 | Mean temperature of warmest quarter |
| Bio 11 | Mean temperature of coldest quarter |
| Bio 12 | Annual precipitation |
| Bio 13 | Precipitation of wettest month |
| Bio 14 | Precipitation of driest month |
| Bio 15 | Precipitation seasonality (coefficient of variation) |
| Bio 16 | Precipitation of wettest quarter |
| Bio 17 | Precipitation of driest quarter |
| Bio 18 | Precipitation of warment quarter |
| Bio 19 | Precipitation of coldest quarter |
