# Supplementary figures and images for: Climatic Niche Shift during Azolla filiculoides Invasion and Its Potential Distribution under Future Scenarios
Source: Plants (Basel). 2019 Oct 18;8(10):424. doi: 10.3390/plants8100424 (PMC6843849; doi:10.3390/plants8100424)

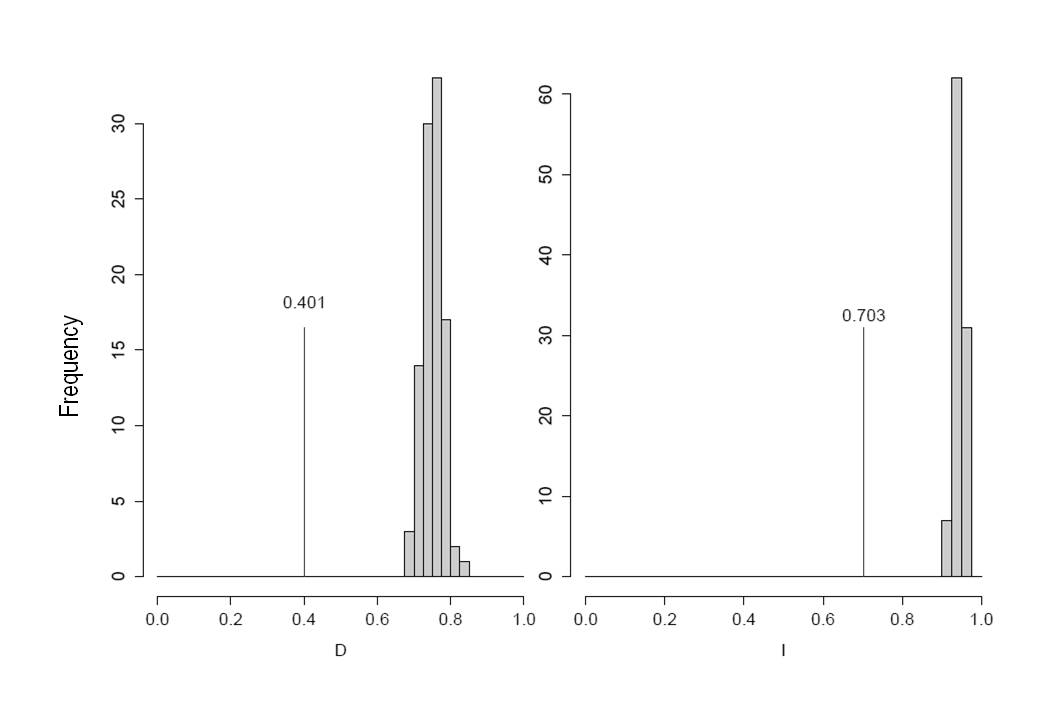

Supplement: Supplementary file 1 [file plants-08-00424-s001.zip › Figure_S1.jpg]

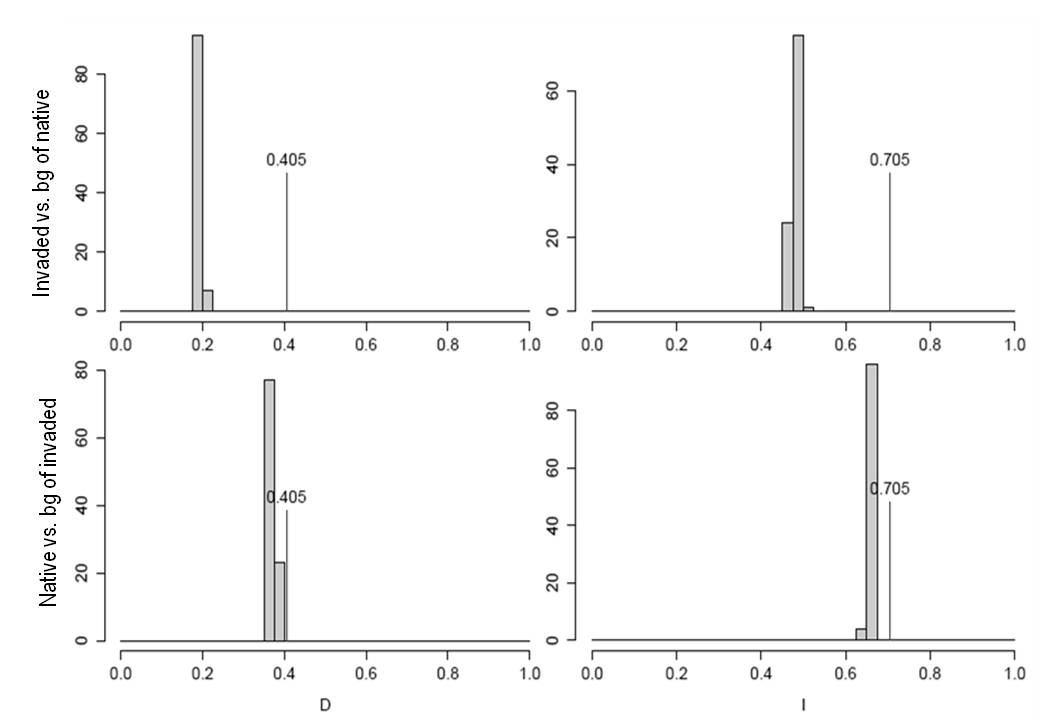

Supplement: Supplementary file 1 [file plants-08-00424-s001.zip › Figure_S2.jpg]

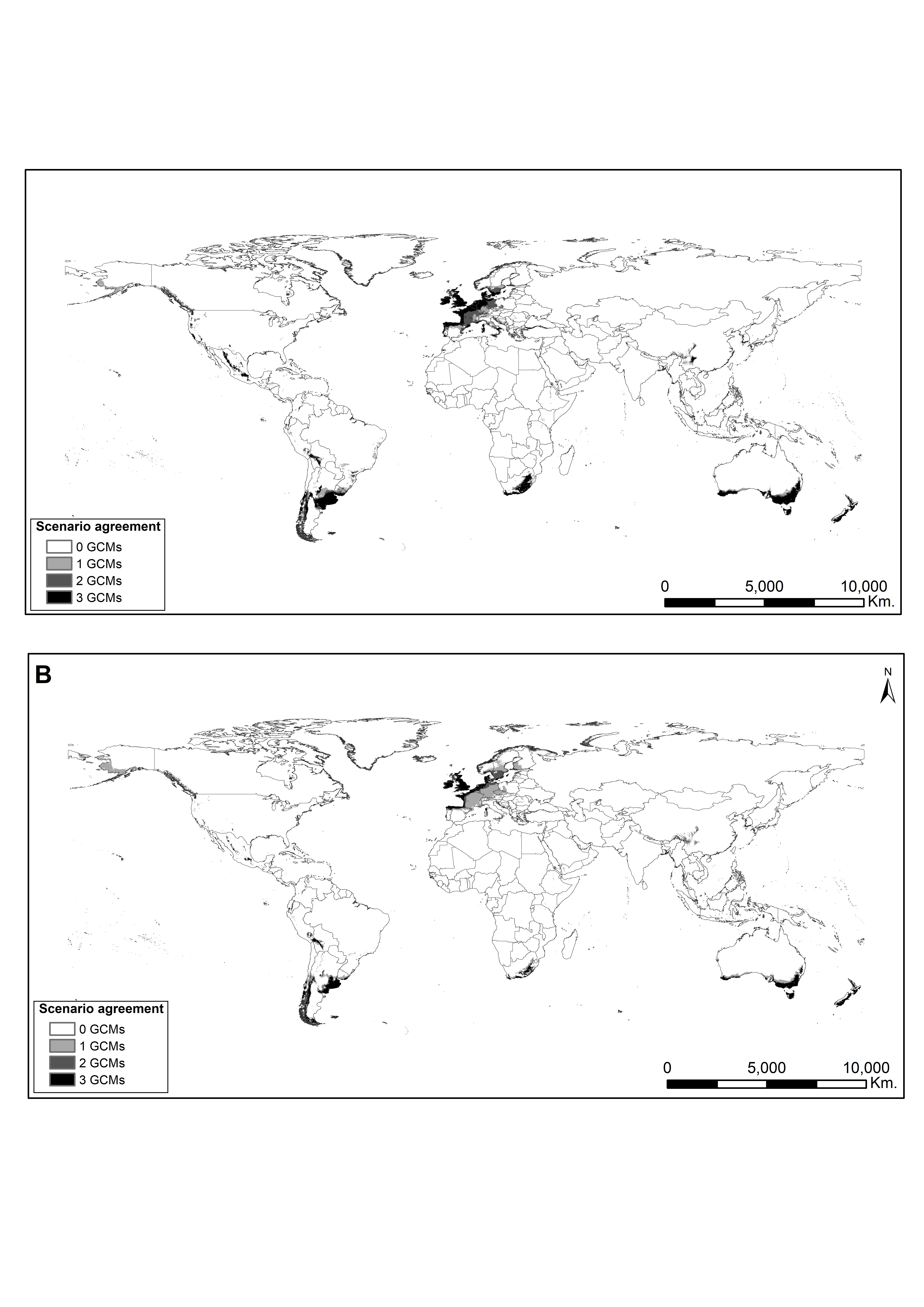

Supplement: Supplementary file 1 [file plants-08-00424-s001.zip › Figure_S3.jpg]

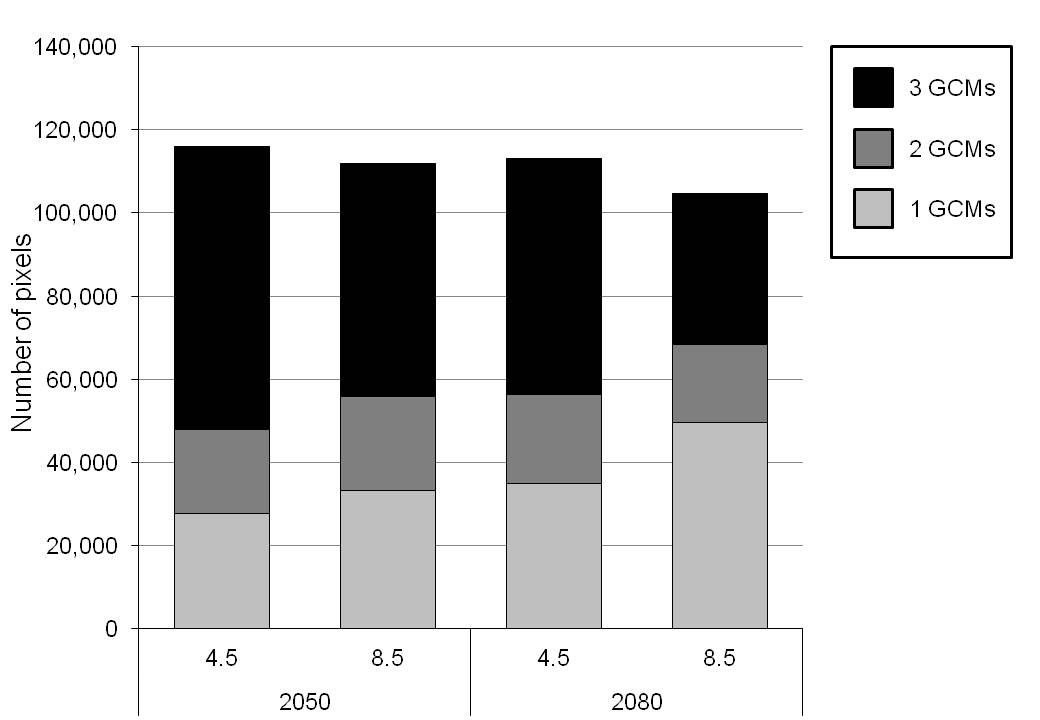

Supplement: Supplementary file 1 [file plants-08-00424-s001.zip › Figure_S4.jpg]
